# The novel choline kinase inhibitor ICL-CCIC-0019 reprograms cellular metabolism and inhibits cancer cell growth

**DOI:** 10.18632/oncotarget.9466

**Published:** 2016-05-19

**Authors:** Sebastian Trousil, Maciej Kaliszczak, Zachary Schug, Quang-De Nguyen, Giampaolo Tomasi, Rosy Favicchio, Diana Brickute, Robin Fortt, Frazer J. Twyman, Laurence Carroll, Andrew Kalusa, Naveenan Navaratnam, Thomas Adejumo, David Carling, Eyal Gottlieb, Eric O. Aboagye

**Affiliations:** ^1^ Comprehensive Cancer Imaging Centre, Department of Surgery and Cancer, Imperial College London, Hammersmith Hospital, London, UK; ^2^ Cancer Research UK, Beatson Institute, Garscube Estate, Glasgow, UK; ^3^ Cellular Stress Group, MRC Clinical Sciences Centre, Faculty of Medicine, Imperial College, Hammersmith Campus, London, UK

**Keywords:** choline kinase, metabolism, mitochondrial function, cancer, positron emission tomography

## Abstract

The glycerophospholipid phosphatidylcholine is the most abundant phospholipid species of eukaryotic membranes and essential for structural integrity and signaling function of cell membranes required for cancer cell growth. Inhibition of choline kinase alpha (CHKA), the first committed step to phosphatidylcholine synthesis, by the selective small-molecule ICL-CCIC-0019, potently suppressed growth of a panel of 60 cancer cell lines with median GI_50_ of 1.12 μM and inhibited tumor xenograft growth in mice. ICL-CCIC-0019 decreased phosphocholine levels and the fraction of labeled choline in lipids, and induced G1 arrest, endoplasmic reticulum stress and apoptosis. Changes in phosphocholine cellular levels following treatment could be detected non-invasively in tumor xenografts by [^18^F]-fluoromethyl-[1,2–^2^H_4_]-choline positron emission tomography. Herein, we reveal a previously unappreciated effect of choline metabolism on mitochondria function. Comparative metabolomics demonstrated that phosphatidylcholine pathway inhibition leads to a metabolically stressed phenotype analogous to mitochondria toxin treatment but without reactive oxygen species activation. Drug treatment decreased mitochondria function with associated reduction of citrate synthase expression and AMPK activation. Glucose and acetate uptake were increased in an attempt to overcome the metabolic stress. This study indicates that choline pathway pharmacological inhibition critically affects the metabolic function of the cell beyond reduced synthesis of phospholipids.

## INTRODUCTION

The majority of neoplasms share the characteristic of a deregulated cellular metabolism to satisfy the diverse demands of biomolecule synthesis required for proliferation [1, 2]. Elevated glucose utilization is clinically routinely assessed by 2-deoxy-2-^18^F-fluoro-D-glucose ([^18^F]-FDG) positron emission tomography (PET) imaging for cancer diagnosis and treatment surveillance. In addition to altered glycolytic handling, lipid metabolism is also frequently deregulated in cancer, particularly in metabolically challenging microenvironments with limited nutrient availability; under such conditions cancer cells activate *de novo* lipogenesis to support growth [3–5]. The synthesized fatty acids serve as membrane components important for the cell's structural integrity and lipid signaling function.

The glycerophospholipid phosphatidylcholine (PC) is the most abundant phospholipid species of eukaryotic membranes and synthesized via the CDP-choline pathway (Supplementary Figure S1A). Upon uptake, choline is phosphorylated via choline kinase (CHK) to phosphocholine (PCho). The high energy donor CDP-choline is then formed from the activating nucleotide cytidine triphosphate (CTP) and PCho by CTP:phosphocholine cytidylyltransferase (CCT). In the final step, CDP-choline:1,2-diacylglycerol cholinephosphotransferase (CPT) catalyzes the formation of PC from CDP-choline and diacylglycerol (DAG). CHK exists in at least three isoforms: CHKA1, CHKA2 and CHKB encoded by two separate genes, of which the A, but not the B isoforms, have been implicated in cancer [6]. While under normal physiological conditions, CCT is the rate-limiting step of the pathway, CHKA is anticipated to be a major regulator in cancer [7, 8].

Aberrant choline metabolic profiles and concomitant CHKA upregulation have been described in most human malignancies, including lung, breast, prostate and endometrial cancer [9–13]. Use of the radiolabeled choline analogue [^11^C]-choline was recently approved by the US Food and Drug Administration as a diagnostic tool for staging of recurrent prostate cancer. Hyperactivated choline metabolism is characterized by elevated intracellular concentrations of PCho and total choline-containing metabolites [14]. The overexpression of CHKA, has been identified to be mainly responsible for this phenotype [7]. CHKA expression is of prognostic significance in clinical breast and lung cancer, where overexpression correlates with disease progression, poor prognosis and reduced survival [9, 15]. Additionally, CHKA has been linked to drug resistance by activating multidrug resistance transporters and to invasiveness [16, 17]. Breast cancer cells exhibit with increasing malignant progression correlatively elevated PCho accumulation [18].

The CDP-choline pathway is distinctly regulated by *de novo* fatty acid biosynthesis. Choline kinase activity is stimulated by growth factors, like epidermal growth factor (EGF) and platelet-derived growth factor (PDGF), as well as oncogenes including Ras, Raf, Mos and c-Src [7]. Although many mitogenic and growth promoting factors result in CHKA, but not CCT, activation, the exact mechanisms remain unclear. Transcriptional silencing of CHKA depletes the intracellular PCho pool [19, 20], which translates into reduced proliferation of MDA-MB-231, MDA-MB-468 and HeLa cells [20, 21] and induction of apoptosis [22]. Furthermore, it promotes differentiation, prevents anchorage-independent growth in HeLa cells and abolishes their ability to form xenografts in athymic mice [20, 21]. Due to its involvement in oncogenic transformation, upregulation in a variety of cancers, and interaction with key signal transduction pathways, CHKA has emerged as a potential target for cancer therapeutics. Previous pharmacological approaches lacked sufficient reporting of specificity, selectivity over other kinases, pharmacokinetics and pharmacodynamics. Furthermore, the effect of CHKA inhibition on metabolism beyond the CDP-choline pathway is unappreciated. Here we elucidate the impact of CHKA inhibition on tumor metabolism using the novel and specific inhibitor ICL-CCIC-0019.

## RESULTS

### ICL-CCIC-0019 is a potent and selective CHKA inhibitor

**Figure 1:**
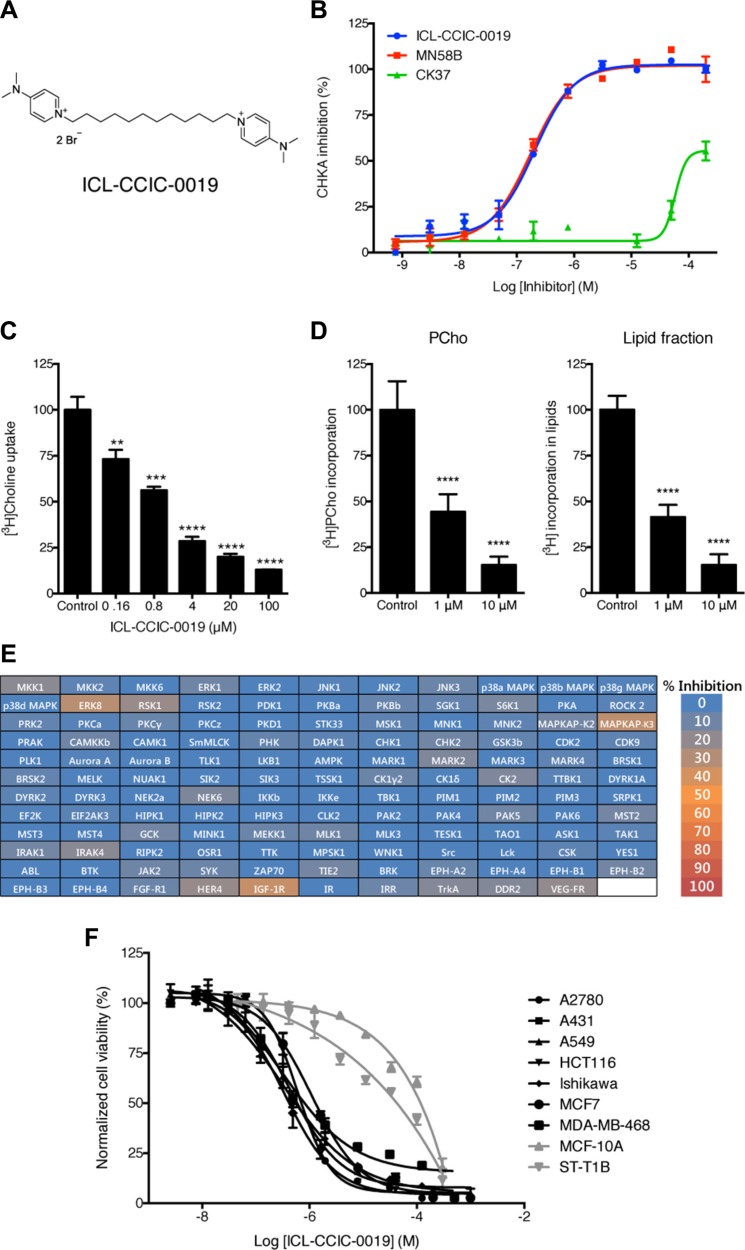
ICL-CCIC-0019 is a selective and potent choline kinase inhibitor (**A**) Structure of ICL-CCIC-0019. (**B**) Enzymatic inhibition of recombinant human Δ49N CHKA2 CHKA against ICL-CCIC-0019, MN58B and CK37. (**C**) [^3^H]Choline uptake in HCT116 cells. Mean of *n* = 3 ± SD; **P ≤ 0.01, ***P ≤ 0.001, ****P ≤ 0.0001. (**D**) Formation of [^3^H]PCho and incorporation of radioactive label into lipids. Mean of *n* = 6 ± SD; ****P ≤ 0.0001. (**E**) Kinase selectivity screen against 131 kinases. (**F**) Antiproliferative activity of ICL-CCIC-0019 against a panel of human cancer (black lines) and normal (grey) cell lines.

Using a focused-library screen, we recently identified a novel small-molecule CHKA inhibitor, ICL-CCIC-0019 (Figure 1A) that is competitive with choline, but not ATP [23]; see this reference for full synthesis of ICL-CCIC-0019 (compound 8 therein). In the present study, we show that the compound inhibits CHKA activity with an IC_50_ of 0.27 ± 0.06 μM and compared with previously described easily accessible CHK inhibitors, is equipotent to MN58B and > 500 times more potent than CK37 (Figure 1B, Supplementary Figure S1B). The substantially less demanding synthesis of ICL-CCIC-0019 compared with MN58B bears additional advantages. ICL-CCIC-0019 dose-dependently reduced the uptake of [^3^H]-choline into HCT116 cells with an EC_50_ of 0.98 ± 0.24 μM (Figure 1C). This consequently diminished intracellular PCho concentration, and importantly, the incorporation of [^3^H]-choline into lipids (Figure 1D). Diminution of [^3^H]-choline labeling of lipids occurred at similar levels to PCho, suggesting that ICL- CCIC-0019 not only depleted the intracellular PCho pool, but also inhibits the synthesis of choline-containing lipids, like PC. To assess kinase specificity, ICL-CCIC-0019 was tested against a panel of 131 human kinases (Figure 1E and Supplementary Table S1). The compound showed low off-target effects as only 5 kinases were inhibited more than 20% at a concentration of 10 μM (≤ 35% in all 131 kinases). Inhibition greater than 20% was attained for insulin-like growth factor 1 (IGF-1R, 35 ± 7% inhibition), mitogen-activated protein kinase-activated protein kinase 3 (MAPKAP-K3, 33 ± 6%), extracellular signal regulated kinase 8 (ERK8, 31 ± 4%), ribosomal S6 kinase 1 (RSK1, 22 ± 6%), and v-erb-a erythroblastic leukemia viral oncogene homolog 4 (HER4, 21 ± 7%).

**Figure 2:**
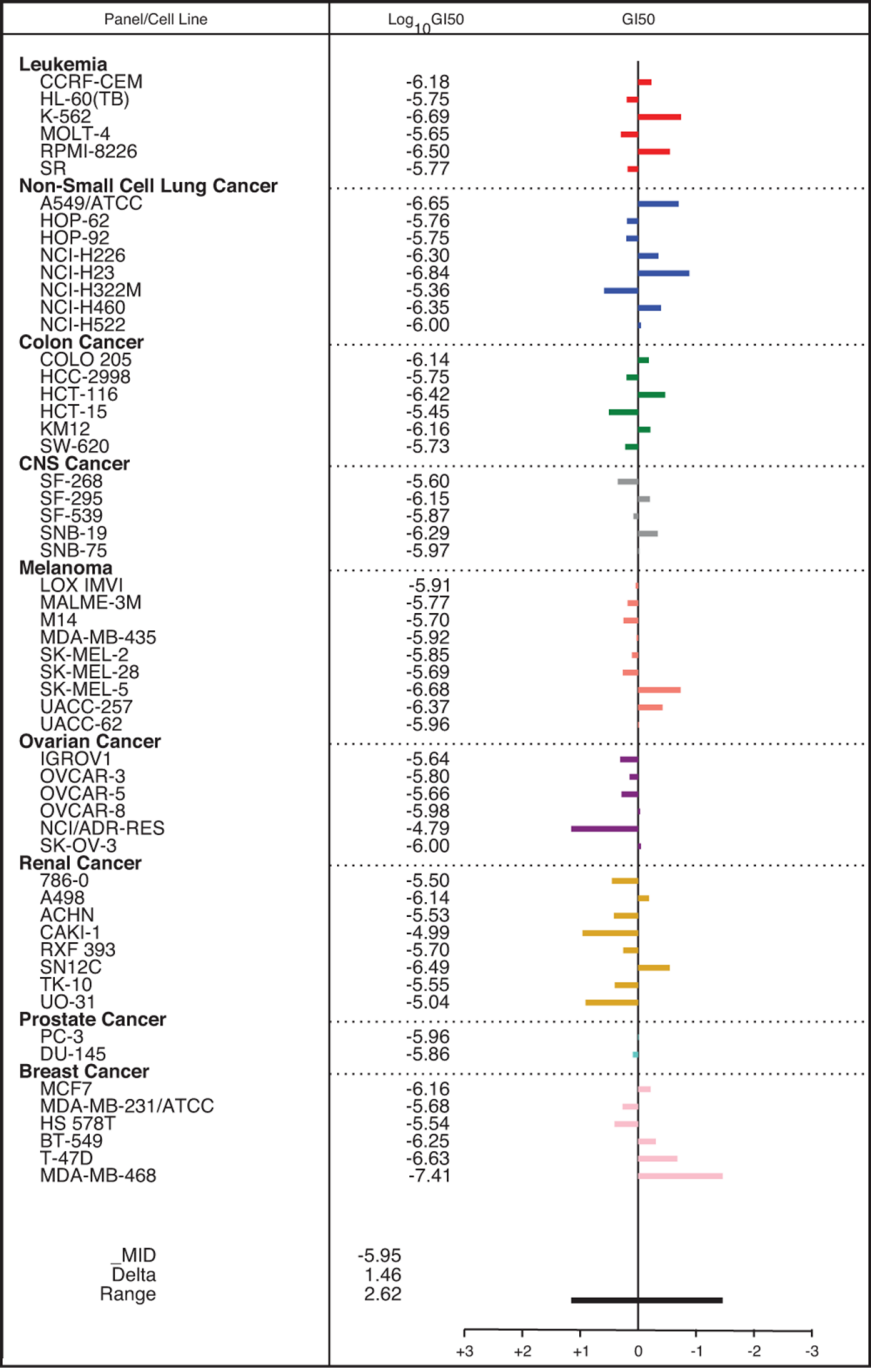
Antiproliferative activity of ICL-CCIC-0019 against the NCI-60 panel of cell lines Mean bar graph and GI_50_ values of all tested cell lines. Bars depict the deviation of individual cell lines from the overall mean value for all the cells tested.

In an initial cell line screen comprising 8 cancer and 2 normal cell lines, cancer-derived lineages were preferentially inhibited with a mean half-maximal growth inhibitory concentration (GI_50_) of 1.09 μM (range 0.38–2.7 μM), while normal cells were insensitive at these concentrations (GI_50_ 30–120 μM; Figure 1F). The compound was then screened against a larger panel of cancer cell lines of diverse histopathologic origin, the NCI-60 screen [24]. The median GI_50_ across all cell lines was 1.12 μM (range 0.0389–16.2 μM, Figure 2). Breast and non-small lung cancer cell lines were most sensitive (median GI_50_ 0.627 and 0.751 μM, respectively), which is line with evidence in the literature of particularly deregulated choline biochemistry in those cancers [9, 10]. Furthermore, the antiproliferative activity of ICL-CCIC-0019 proved to be equipotent to MN58B and superior to CK37 (Supplementary Figure S1C). Mining of publicly available Cancer Genome Atlas (TCGA) expression data identified *CHKA* amplification and/or mRNA upregulation in 11% of breast, 5–10% of lung, 8% of ovarian, 7% of colorectal and 5% of prostate cancers. Furthermore, CHKA mRNA expression is higher in multiple tumor types compared to normal tissue (Supplementary Figure S1D). The gene was rarely mutated or deleted (0–2.5% mutation rate and < 1% deletion among all tested cancer types). The consistently high expression in a wide variety of tumor types highlights the relevance of CHKA in malignancy and role as a therapeutic target.

Knockdown of CHKA by shRNA produced similar biochemical and growth inhibitory effects as ICL-CCIC-0019 (Supplementary Figure S1E–S1H). Due the bolaamphiphilic character of the ICL-CCIC-0019 compound class, comparable to surfactants, we investigated the potential of the compound to non-specifically lyse cells. Surfactant-induced cell lysis triggers formation of pores in the cell membrane and subsequent release of cytosolic proteins, such as lactate dehydrogenase (LDH), into the supernatant [25]. Incubation of HCT116 cells with 1–25 μM ICL-CCIC-0019 did not elevate LDH concentration in the supernatant (Supplementary Figure S1I). Furthermore, enzyme dimerization, which is required to exert CHKA activity, was not compromised by treatment with ICL-CCIC-0019 (Supplementary Figure S1J), inferring that the mechanism of action did not involve destabilization of CHKA complexes.

### CHKA inhibition arrests cell cycle and causes ER stress response

**Figure 3:**
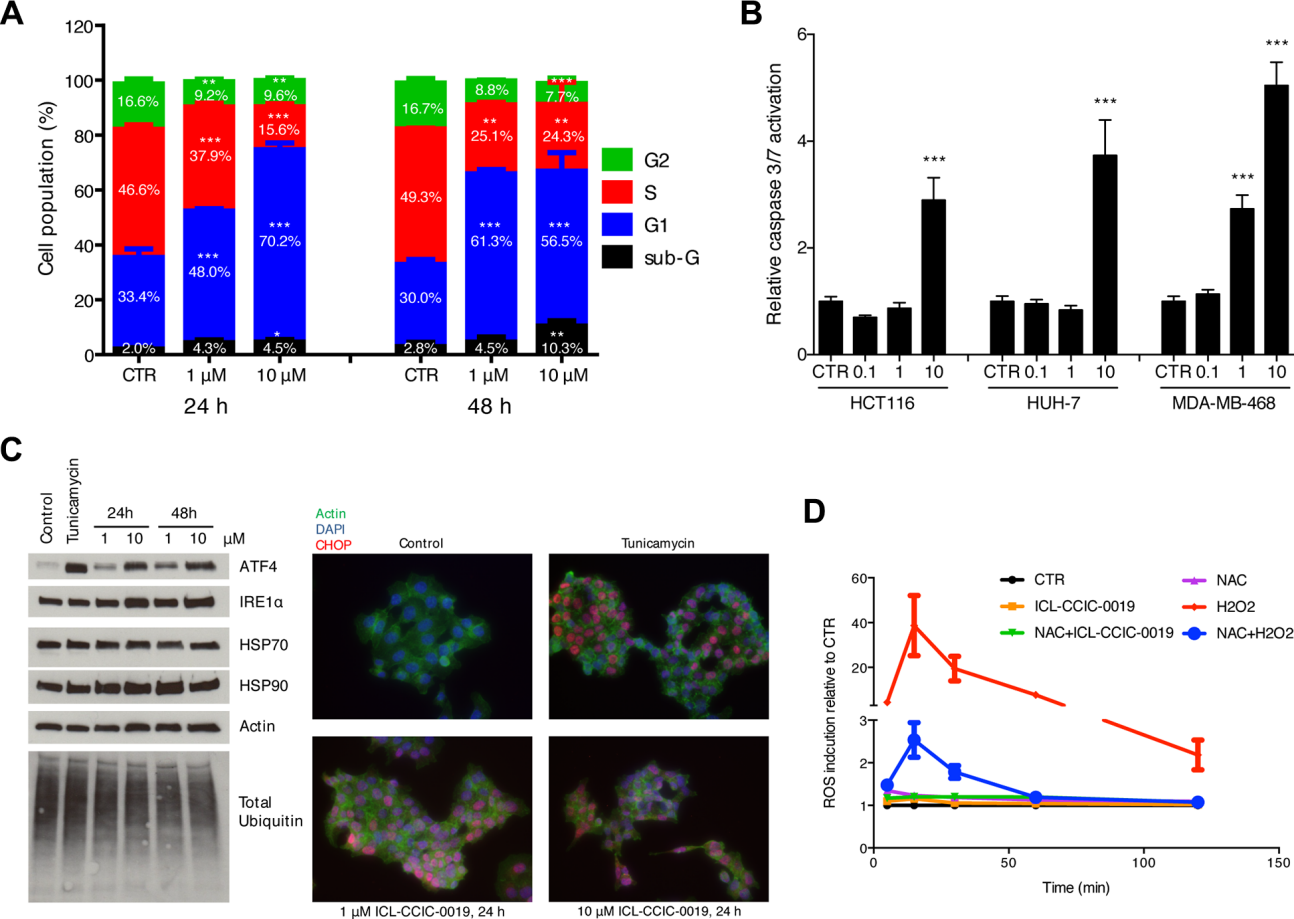
ICL-CCIC-0019 induces G1 arrest, increases sub-G1 population and causes ER stress (**A**) HCT116 cells were incubated with inhibitor for 24 or 48 hours and cell cycle populations quantified by flow cytometry using propidium iodine staining. (**B**) HCT116 cells were incubated with indicated doses in μM of ICL-CCIC-0019 for 48 hours and caspase 3/7 activity determined by caspase Glo assay and normalized to protein. Data are mean of *n* = 3 ± SD; ***P ≤ 0.001. (**C**) Analysis of key regulators of ER stress in HCT116 cells by western blot and immunofluorescence. Tunicamycin (2 μg/mL for 4 hours) was used as positive control. (**D**) Formation of reactive oxygen species in HCT116 cells upon ICL-CCIC-0019 treatment. CTR, control, NAC, N-acetyl cysteine, Data in A, B and D are expressed as mean of *n* = 3 ± SD; *P ≤ 0.05, **P ≤ 0.01, ***P ≤ 0.001.

ICL-CCIC-0019 dose-dependently arrested cells in the G1 phase of the cell cycle after 24 h of treatment (2-fold increase at 10 μM) and increased the sub-G1 population, representing apoptotic and dead cells, 3.7-fold after 48 h (Figure 3A). Caspase-3/7-mediated apoptosis was seen following 48 h treatment with varying concentrations of ICL-CCIC-0019 in HCT116, HUH-7 and MDA-MB-468 cells (Figure 3B). Depletion of PC was previously shown to induce ER stress [26]. Therefore, the expression of ER stress response markers was investigated. ICL-CCIC-0019 dose- and time-dependently increased the expression of ATF4, a transcription factor for target proteins that resolve ER stress, and to lesser extent IRE1α (Figure 3C). In keeping with above, expression and nuclear localization of the transcription factor CHOP was higher in ICL-CCIC-0019 treated cells independent of protein ubiquitination and HSP70 expression. Moderate increases in HSP90 expression might have further contributed to the ER stress phenotype. Notably, ER stress was not accompanied by formation of reactive oxygen species (ROS, Figure 3D).

### ICL-CCIC-0019 inhibits CHKA *in vivo* and has potent antitumor activity

**Figure 4:**
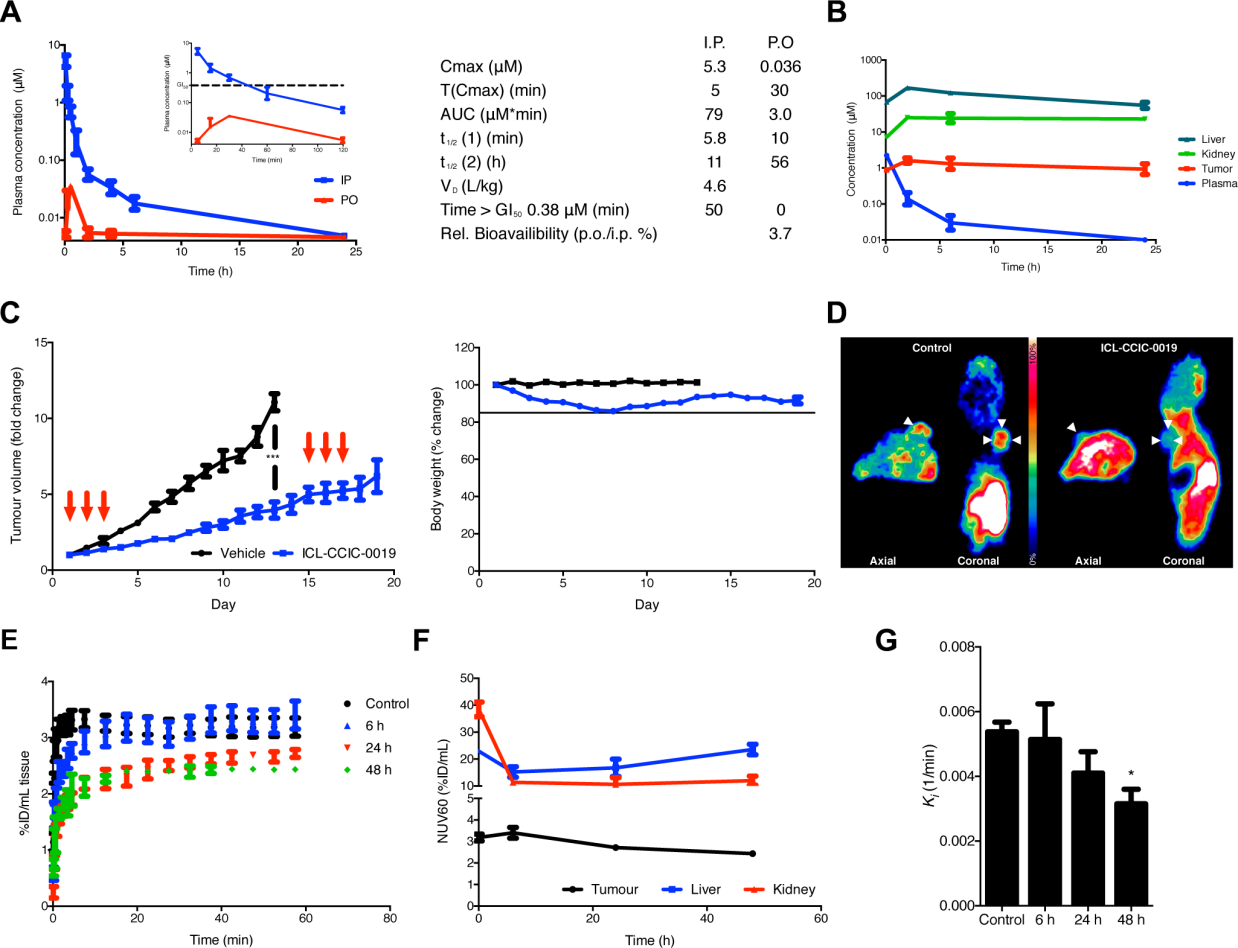
Plasma and tissue pharmacokinetics of ICL-CCIC-0019 (**A**) BALB/c mice were administered 10 mg/kg ICL-CCIC-0019 p.o. or i.p. and plasma obtained 5, 15, 30, 60 minutes and 2, 4, 6 and 24 hours post injection. Figure insert displays first 120 minutes only. Plasma pharmacokinetic variables are shown. (**B**) Tissue distribution of ICL-CCIC-0019. HCT116 xenograft bearing BALB/c nude mice were treated with 10 mg/kg ICL-CCIC-0019 and plasma, tumor, liver and kidney inhibitor concentrations determined at indicated time points. (**C**) Antitumor activity of ICL-CCIC-0019 and body weight changes in a HCT116 xenograft model. Arrows indicate time of dosing. (**D**) Representative PET image using the target-competitive probe [^18^F]-D4-FCH in ICL-CCIC-0019-treated HCT116 xenografts. (**E**) TACs of dynamic PET imaging scans. (**F**) Tumor, liver and kidney-associated radioactivity upon ICL-CCIC-0019 treatment. (**G**) The pharmacokinetic macro rate constant for net irreversible uptake, *K*_i_, was computed employing a two-tissue irreversible compartmental model. Data represent mean of *n* = 4–5 per group ± SEM; *P ≤ 0.05.

We selected the HCT116 xenograft model for further work. HCT116 cells have intermediate sensitivity to ICL-CCIC-0019 and we have previously shown that the model is sensitive to inhibitory activity of other drugs on [^18^F]-D4-FCH tumor uptake [27]. Plasma pharmacokinetic profiles were established in BALB/c mice following a single injection of 10 mg/kg ICL-CCIC-0019. ICL-CCIC-0019 was rapidly cleared following i.p. administration and plasma concentrations above the GI_50_ of HCT116 cells were maintained for ca. 50 minutes (Figure 4A). Analysis of plasma indicated that the compound was metabolically stable (Supplementary Figure S2A) although its oral bioavailability was limited. BALB/c nude mice bearing HCT116 xenografts were employed for analysis of tumor, liver, kidney and plasma concentrations (Figure 4B). The compound was rapidly extracted by the investigated tissues and tumor concentrations above the GI_50_ were maintained throughout the study (Figure 4B). Extensive accumulation in liver and kidneys were observed, although clearance from liver was more rapid than from tumor. In HCT116 xenograft bearing mice, treatment with ICL-CCIC-0019 on days 1–3 with 5 mg/kg i.p. once a day followed by an 11 day recovery period (consistent with the inhibitor's pharmacokinetics) resulted in potent antitumour activity (Figure 4C). To determine whether ICL-CCIC-0019 could attenuate CHKA activity *in vivo*, [^18^F]-D4-FCH PET imaging was performed. The radiotracer and inhibitor target the same protein, thus permitting non-invasive assessment of enzyme activity (pharmacodynamics) in the physiological and complex milieu of the tumor, as well as normal tissues. A marked reduction in radiotracer uptake was observed in ICL-CCIC-0019-treated HCT116 xenografts at 24 and 48 hours following a i.p. single dose of 10 mg/kg, compared to the vehicle-treated cohort [AUC_0–60_ control: 191.9 (%ID/mL)*min; AUC_0–60_ 24 hours: 145.8 (%ID/mL)*min; AUC_0–60_ 48 hours: 139.4 (%ID/mL)*min; Figure 4D–4E]. Interestingly, despite the rapid tissue distribution, whereby ICL-CCIC-0019 concentrations in the tumors peaked at 2 hours post injection, PET imaging at 6 hours post ICL-CCIC-0019 administration did not result in significant reduction of [^18^F]-D4-FCH uptake [AUC_0–60_ 6 hours: 187.7 (%ID/mL)*min]. While cardiac pharmacodynamics were not influenced by treatment, liver and kidney radiotracer retentions were diminished, comparable to tumor data (Figure 4F and Supplementary Figure S2B).

We employed kinetic modeling to further characterize ICL-CCIC-0019-mediated choline kinase inhibition *in vivo*. Whereas normalized tissue uptake values reflect tracer concentration in a 3-dimensional region of interest, kinetic modeling accounts for tissue uptake relative to plasma. A two-tissue irreversible compartmental model, as previously reported, was employed with the hypothesis that *k*_3_ would represent the rate by which the tracer is phosphorylated by choline kinase [28], (Supplementary Figure S2C). Although this enzymatic reaction is in principle reversible, it was assumed that the high dependency of cancer cells to incorporate choline made it unlikely that catalytic hydrolysis of PCho prevails. Best fits and more physiological parameters were obtained with a 3k (excluding *k*_4_) instead of a 4k model. ICL-CCIC-0019 treatment significantly decreased tumor *K*_1_, denoting the flux from blood to tissue, at all investigated time points (73% and 75% decrease compared to control after 24 and 48 hours, respectively; Supplementary Figure S2D) and to a lesser extent in kidneys and liver (Supplementary Figure S2D). The opposing flux from tissue into the blood stream, *k*_2_, was slightly decreased by treatment in tumor (Supplementary Figure S2D). Interestingly, the micro rate constant *k*_3_, which represents intracellular radiotracer trapping, was non-significantly altered (Supplementary Figure S2D). In consequence to these micro parameter changes, the macro parameter *K*_i_ denoting the net irreversible uptake rate was significantly decreased in tumor after 48 hours (Figure 4G and Supplementary Figure S2D); changes in kinetic flux were predominantly driven by alterations in *K*_1_. In all examined tissues, the blood volume *V*_b_ decreased initially after treatment (Supplementary Figure S2D). These perfusion deficits were more pronounced and persistent in tumor compared to liver and kidney. To rule out that these kinetic changes are solely due to choline transport inhibition, we analyzed the choline metabolites of [^18^F]-D4-FCH in HCT116 cells treated with ICL-CCIC-0019. As expected for CHKA inhibition, ICL-CCIC-0019 decreased the [^18^F]-D4-PCho peak, changing the relative ratio of [^18^F]-D4-Cho:[^18^F]-D4-PCho from 72:28 in the vehicle control to 87:13 in the treatment group (Supplementary Figure S2E–S2F).

These data support that ICL-CCIC-0019 is a specific CHKA inhibitor, capable of inhibiting its target *in vivo* resulting in potent antitumor activity.

### Effect of CHKA inhibition on soluble cellular metabolites

**Figure 5:**
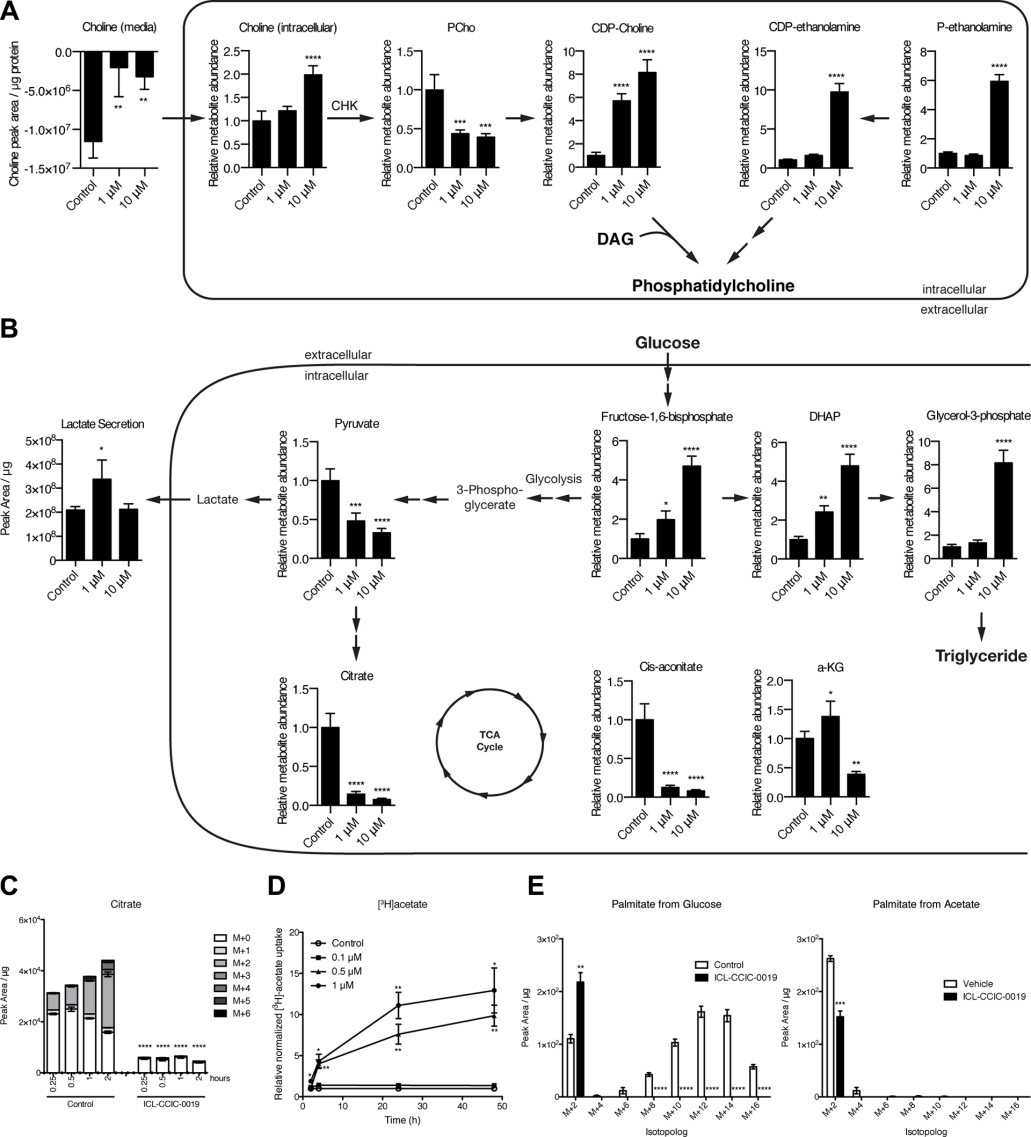
ICL-CCIC-0019 inhibits PC biosynthesis and increases glycolysis Steady-state global metabolomics analysis of HCT116 cells treated with ICL-CCIC-0019 for 24 hours depicting CDP-choline and ethanolamine kinase pathway (**A**) and glycolysis, and TCA cycle intermediates (**B**). (**C**) HCT116 cells were treated with ICL-CCIC-0019 for 24 hours and subsequently subjected to ^13^C-labeled glucose for indicated times. Intracellular citrate is shown. (**D**) [^3^H]acetate uptake upon ICL-CCIC-0019 treatment in HCT116 cells. (**E**) ^13^C-glucose and ^13^C-acetate-derived isotopomer distributions of palmitate. Mean of *n* = 4 ± SD; *P ≤ 0.05; **P ≤ 0.01, ***P ≤ 0.001, ****P ≤ 0.0001.

We then investigated the metabolic consequences of CHKA inhibition by ICL-CCIC-0019 using liquid chromatography-mass spectrometry (LC-MS) of soluble metabolites. CHKA inhibition expectedly decreased the uptake of choline from the media and potently depleted the intracellular PCho pool (−56% at 1 μM and −61% at 10 μM for 24 h, Figure 5A). Unexpectedly, given the reduction in ^3^H-choline incorporation into lipids (Figure 1D), CDP-choline was dose-dependently higher after inhibitor treatment (Figure 5A). CDP-ethanolamine pathway intermediates (phosphoethanolamine and CDP-ethanolamine), as well as precursors of diacylglycerol required for PC synthesis such as glycerol-3-phosphate and dihydroxyacetone phosphate (DHAP) were also increased following ICL-CCIC-0019 treatment (Figure 5B) suggesting inhibition or reduced substrate availability for processes culminating in PE and PC synthesis, independent of PCho inhibition. PCho is affected by phospholipid breakdown. In the HCT116 cell line, however, NMR-detectable glycerophosphocholine (GPC) levels were below the level of quantification (LoQ) relative to PCho; GPC levels were still undetectable 2 h after treatment with ICL-CCIC-0019 but increased at 24 h to levels just above the LoQ (data not shown) indicating the GPC pathway plays a minor role in this setting.

Treatment with ICL-CCIC-0019 markedly decreased citrate and cis-aconitate at 1 and 10 μM, and alpha-ketoglutarate at the high concentration (Figure 5B). Succinate and malate were unaffected (data not shown). This prompted us to examine in more detail the changes in metabolism to overcome the TCA cycle defect. The resulting energy crisis led to increases in the glycolytic intermediate fructose-1,6-bisphosphate (Figure 5B). Low AMP/ATP ratio allosterically stimulates glycolysis through the rate limiting enzyme phosphofructokinase. We observed an increased [^18^F]-FDG uptake (Supplementary Figure S3A) indicating enhanced glucose flux. Uniformly ^13^C-labeled glucose flux studies confirmed increased glycolysis, as shown by increased glucose-6-phosphate and glyceraldehyde-3-phosphate (Supplementary Figure S3B), as well as diminished TCA cycle activity (Figure 5C). The pentose phosphate pathway intermediate ribose phosphate was increased upon ICL-CCIC-0019 (Supplementary Figure S3C), however, pathways that branch off glycolysis, as for example serine synthesis pathway, did not show increased labeling with ^13^C-glucose and elevated unlabeled serine suggested enhanced utilization of exogenous serine (Supplementary Figure S3D). Serine can condense with palmitate to make 3-keto-dihydrosphingosine, a precursor of *de novo* ceramide synthesis previously implicated as a marker of choline kinase inhibitor MN58B [29]. Furthermore, serine can be a carbon donor to *S*-adenosylmethionine (SAM) in two ways; either as a methyl donor via the tetrahydrofolate (THF) cycle during methionine salvage or as a carbon donor to the adenosyl moiety of SAM during nucleotide biosynthesis (via glycine and the THF cycle) [30]. The ICL-CCIC-0019 concentration-dependent uptake and intracellular buildup of serine was found to be associated with a 11% increase in SAM and 26% decrease in *S*-adenosylhomocysteine (SAH) perhaps suggesting an increase in phosphatidylethanolamine *N*-methyltransferase activity to supply PC from phosphatidylethanolamine (PE; rather than PE to phosphatidylserine using serine; thus serine buildup) under the nutrient stress associated with reduced PC synthesis. It is interesting to note that the rate of serine biosynthesis was comparable in vehicle and ICL-CCIC-0019 treated cells (Supplementary Figure S3E) while the biosynthesis of many other non-essential amino acids including aspartate and glutamate was strongly inhibited (Supplementary Figure S3F–S3G). This suggests that cells expedite serine biosynthesis for SAM production and downstream PC generation from PE or even ceramide production.

Furthermore, *de novo* fatty acid synthesis was inhibited and neither glucose nor acetate served as substrates, despite a 10-fold increased acetate uptake upon ICL-CCIC-0019 treatment (Figure 5D–5E). Most of the labeled acetate was incorporated into acetyl-carnitine (Supplementary Figure S3H). Of interest, medium chain carnitine esters and palmitoycarnitine were markedly increased (Supplementary Figure S3I) indicating an inability of the mitochondria to use medium chain esters derived from peroxisomes. Thus, the dysfunctional TCA cycle and consequent ER stress appear to increase glucose uptake, however, lack of possibilities to branch into other pathways result in almost quantitative secretion as lactate (Supplementary Figure S3J). The lack of glutamine uptake under these conditions (Supplementary Figure S3J) is in keeping with a dysfunctional TCA cycle/mitochondria. While palmitate M+2 from glucose was higher in drug treated samples, there was a stark reduction in other isotopologues (M+4, M+6, M+8, M+10, M+12, and M+14). Restoration of naturally occurring M+2 from palmitate when labeling ceases, or unperturbed elongase activity (mediating addition of glucose-labeled acetyl-CoA unit to pre-existing myristate) could have led to the changes in palmitate M+2 seen.

### Mitochondrial dysfunction and AMPK as downstream targets of choline kinase inhibition

**Figure 6:**
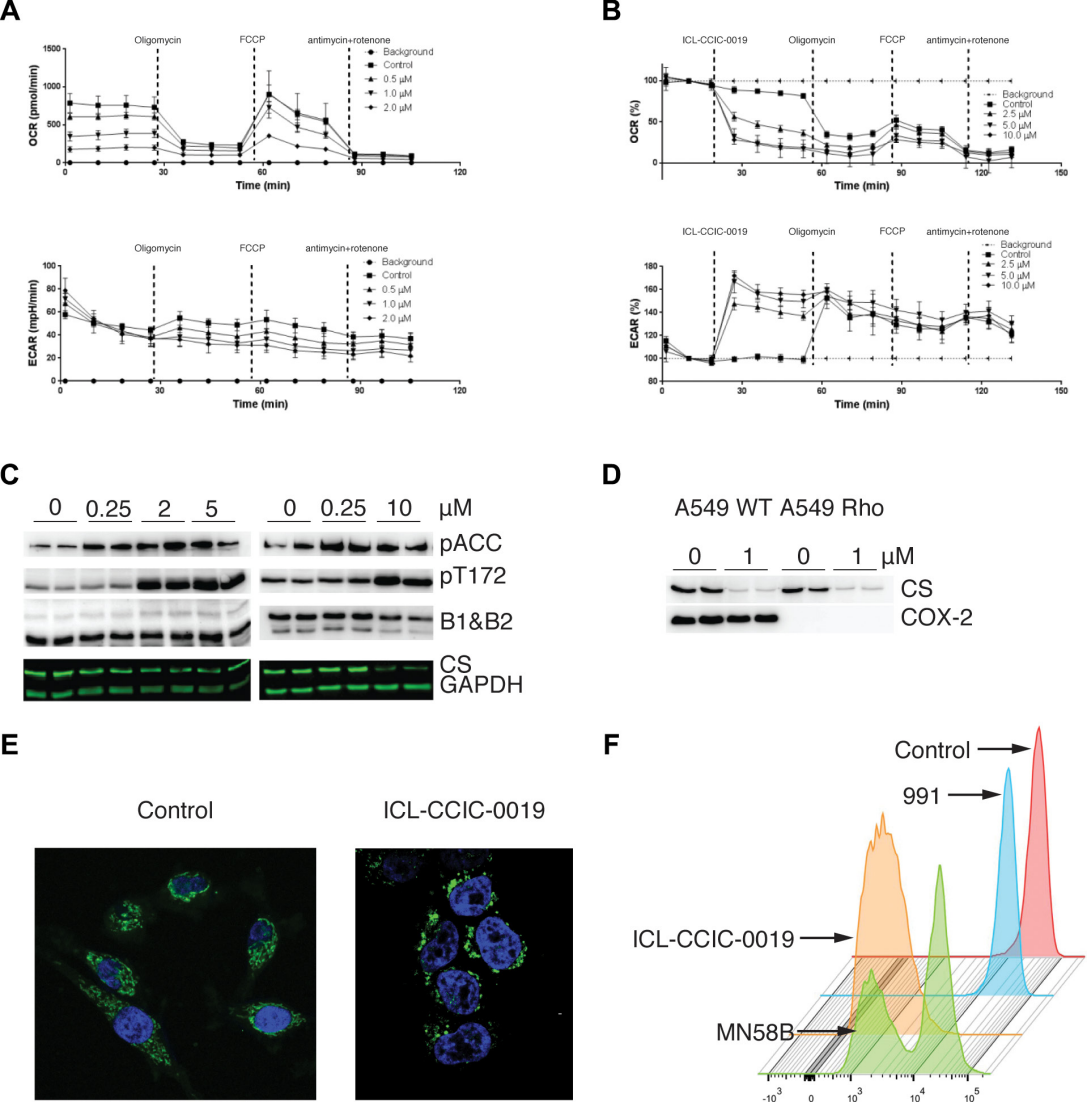
Mitochondria dynamic changes upon CHK inhibition Oxygen consumption rate (OCR) and extracellular acidification rate (ECAR) of HCT116 cells following 24 h pre-treatment with ICL-CCIC-0019 (**A**), and following direct addition of ICL-CCIC-0019 (**B**). Vertical lines indicate the time of injection of ATP synthase inhibitor oligomycin (0.3 μg/mL), the pharmacological uncoupler FCCP (0.6 μM), complex III inhibitor antimycin (5 μg/mL), and complex I inhibitor rotenone (5 μg/mL) and ICL-CCIC-0019 (1 μM). (**C**) Effect of ICL-CCIC-0019 on AMPK activation in HCT116 cells; phosphorylated acetyl-coA carboxylase (pACC), AMPK phosphorylation at Thr172 (pT172), AMPK beta 1 and 2 subunits (B1 & B2) and citrate synthase (CS). (**D**) Effect of ICL-CCIC-0019 on citrate synthase in A459 WT and isogenic A549 Rho cells. (**E**) Effect of ICL-CCIC-0019 (5 μM, 24 h) on mitochondrial networks. Cells were stained with anti-mitochondrial antibody (green) and DAPI (blue). (**F**) Effect different inhibitors (10 μM each for 24 h) on mitochondria membrane potential measured by the tetramethylrhodamine ethyl ester dye method.

A number of observations above led us to further examine a previously unappreciated involvement of mitochondria in the mechanism of action of choline kinase inhibition. Seahorse assays confirmed a dose-related suppression of mitochondrial respiration at 24 h after inhibitor treatment (Figure 6A, 6B). In the presence of glucose in medium, ICL-CCIC-0019 decreased mitochondrial oxygen consumption (basal, ATP production, maximum respiration, spare capacity and proton leak) in a drug concentration related manner. Interestingly, ICL-CCIC-0019 and MN58B decreased mitochondrial respiration immediately after adding inhibitor (acute effect; Supplementary Figure S4A, S4B). The marked inhibition of TCA cycle without increased ROS, suggested a non-classical mitochondrial toxin effect. Against the background of energy stress we examined AMPK substrates. Both phospho-acetyl-CoA carboxylase (pACC) and AMPK pT172 increased with CHKA inhibitor treatment in a concentration dependent manner in support of AMPK activation (Figure 6C); total ACC was not sensitive to different drug concentrations (Supplementary Figure S4B). Cell-free kinase assays indicated that ICL-CCIC-0019 did not directly alter AMPK activity (Figure 1E), while the inhibitor increased the AMP/ATP ratio (Supplementary Figure S4C). Thus the observed effect is indirect, likely via sensing of energy stress [31].

We showed that the reduction in citrate following CHKA inhibition was associated with drug concentration related decrease in citrate synthase expression in HCT116 cells at 24 h after treatment (Figure 6C). Citrate synthase (CS), the first rate-limiting enzyme in the TCA cycle is critical for mitochondrial respiration. Knockdown of the protein stimulates glycolysis [32] in keeping with the observations of increased glucose flux seen above. Reduction in the nuclear encoded CS expression is also a marker of mitophagy, the selective removal of mitochondria through autophagy (for review see [33]). Disruption of mitochondrial function such as treatment with electron transport chain inhibitors or depletion of mtDNA does not generally affect CS enzyme expression or activity, making it a useful semi-quantitative indicator of mitochondria mass in cells. To confirm specificity of CS expression in this context we verified the effect of CHKA inhibition in A549 human lung cancer cells and isogenic cells with depleted mtDNA (A549 *Rho*). In these cells ICL-CCIC-0019 diminished CS expression irrespective of mtDNA status in comparison with COX2 (Figure 6D). Mitochondria are highly dynamic organelles that exist in networks. Fluorescence microscopy of mitochondria stained with anti-mitochondrial antibody distinctly showed disruption of mitochondrial networks in cells treated with ICL-CCIC-0019 (Figure 6E). Furthermore tetramethylrhodamine, ethyl ester (TMRE)-detectable mitochondrial membrane potential decreased with ICL-CCIC-0019 or MN58B treatment (Figure 6F) compared to vehicle control or negative control (small molecule AMPK activator, 991 [34]).

## DISCUSSION

The glycerophospholipid PC is the most abundant phospholipid species of eukaryotic membranes and essential for structural integrity and signaling function of cell membranes required for cancer cell growth [35]. CHKA represents the first committed step to PC synthesis. The protein is overexpressed in several cancers through amplification or mRNA upregulation. In cancer cells, overexpression of CHKA is associated with transformation of normal cells to cancer [18], and conversely, depletion of CHKA by RNAi can reduce cell survival and tumor growth (Supplementary Figure S1C and[8, 20, 36]). To date, attention in the field of cancer has focused on the requirement of choline together with channeling of fatty acids into PC synthesis to support the increased need for membrane synthesis; however, this mechanism alone does not explain the effects of chemical inhibitors. CHKA chemical inhibitors developed to date phenocopy CHKA knockdown showing a decrease in PCho levels, however, only minimal cytotoxicity is seen with ATP-site binding, relative to choline-site or mixed ATP-site/choline-site inhibitors, leading to the suggestion that a CHKA-independent or non-catalytic role for CHKA is important for promoting cell survival [37]. We report the pharmacology of a new CHKA inhibitor, and demonstrate that CHKA chemical inhibition can induce profound ER stress response and inhibit mitochondrial respiration as additional potential mechanisms for reduced survival.

As a starting point, we focused on chemical inhibition of CHKA, using the selective choline-site specific CHK small-molecule ICL-CCIC-0019 [23]. Notably, structural similarities exist between the *N, N*-dimethylaminopyridine moiety employed as a cationic choline-mimicking moiety in ICL-CCIC-0019 [23] and nature identical lipids such as C_2_/C_6_-ceramides, lyso-phosphatidic acid and lyso-PC. ICL-CCIC-0019 was highly specific in a multi-kinase screen, decreased PCho levels together with the fraction of labeled choline in lipids (mainly PC), and induced G1 arrest, endoplasmic reticulum stress and apoptosis. We measured incorporation of [^3^H]-choline into lipids and assumed therefore that the diminution of the lipid labeled fraction is due to reduction of phosphatidylcholine. It should be noted, however, that [^3^H]-choline can be metabolized and incorporated into other lipids including phosphatidylethanolamine or phosphatidylserine, although these alternative mechanisms are likely to be limited compared to phosphatidylcholine. Phosphoethanolamine levels were not decreased upon drug treatment, suggesting that ICL-CCIC-0019 did not inhibit ethanolamine kinase. Reduction in cell survival occurred across 60 cell lines of diverse origin with median GI_50_ of 1.12 μM and the compound also inhibited colon cancer growth in mice.

Based on our data, we assert that the ability of ICL-CCIC-0019 to effect rapid and sustained reductions of CDP-choline pathway, precluding significant cellular adaptation to replenish phosphatidylcholine, likely leads to reduction in proliferation seen across several cancer cell lines including the NCI-60 panel. It should be noted, however, that using a novel CHKA inhibitor, V-11–0711 [37], two independent laboratories have shown that drug-induced reductions of steady state PCho occurring after 24 h in HeLa and MDA-MB-231 were not sufficient to decrease cell viability, compared to CHKA RNAi [37, 38]; cell viability was lost only in SUM149 cells at similar drug concentrations. The groups infer that it is the depletion of CHKA protein - acting through non-catalytic functions [39] - but not inhibition of CHKA catalytic activity that is able to decrease cell survival. This conclusion, supported by the insensitivity of catalytically-inactive mutant CHKA2 D306A transformed cells to EGF-stimulated growth [39], contrasts the substantial literature demonstrating anti-proliferative effect of diverse small molecule CHKA ATP-, choline- and mixed-site binders on cell viability under unrestricted-growth media conditions and in mice [29, 40–44]. The hypothesis neither incorporates drug resistance possibilities, nor importantly the ability of the compounds to rapidly deplete phosphatidylcholine without significant regeneration. Thus, while non-catalytic (‘scaffold’) functions of CHKA exist [39], and loss of the PCho generation step alone may be insufficient to decrease cell viability under certain conditions, we cannot rule out that small molecule inhibitors can affect cell viability via the CDP-choline pathway due to rapid depletion of dynamic membrane phosphatidylcholine synthesis required for cellular integrity and mitochondria function.

Impaired choline transport and phosphorylation following treatment was detected non-invasively in tumor and liver by whole body [^18^F]-D4-FCH. The time-dependent reduction in flux constant *K*_i_ of [^18^F]-D4-FCH occurred as a result of drug accumulation in tissue and inhibition of CHK, and was associated with the tumor growth inhibition observed. The rapid uptake of labeled choline tracers, together with limited efflux back into the blood pool renders kinetic models unable to independently distinguish between *K*_1_ and *k*_3_; the macro-parameter *K*_i_ better reflects retention than *k*_3_ [28, 45]. Both *K*_1_ and *K*_i_ decreased with treatment suggesting that ICL-CCIC-0019 inhibits choline extraction (transfer from blood to tissue) and retention. Indeed *in vitro* studies showed that ICL-CCIC-0019 competes with a labeled choline tracer for uptake (Supplementary Figure S2E–S2F). At this stage, we notionally attributed the reduction in PC seen *in vitro* to reduction in PCho. We investigated whether a non-catalytic modulation of CHKA may be responsible for the reduced cell survival seen. Alteration in CHKA dimerization was unaffected by ICL-CCIC-0019 thus destabilization of the protein was ruled out. Induction of endoplasmic reticulum stress also led us to investigate ROS formation; ROS formation was ruled out as a mechanism of action.

We then resorted to metabolomics to identify additional mechanisms. We confirmed the reduction in PCho production and concomitant increases in free intracellular choline. CDP-Cho pool-size increased dose-dependently with drug treatment indicating blockade of CPT. This observation was unexpected, and while consistent with our finding of reduced labeled choline incorporation into PC, could also be explained by activation of CCT. Further work is required to clarify the specific enzymes inhibited. The reduction in PC could also have resulted from reductions in fatty acids or DAG. In our studies we observed specific decreases in the following fatty acids: palmitoleic, oleic and dodecanoic acids, which could have contributed to the reduction in PC synthesis. DAG levels were not measured. Interestingly, the analogous CDP-ethanolamine:1,2-diacylglycerol ethanolaminephosphotransferase (EPT) was also inhibited but only at higher drug concentration. Very few inhibitors of CPT/EPT have been reported, and indeed, these two enzymes were not included in our selectivity screen. In their study, Bladergroen and co-workers noted that C_2_-ceramides selectively inhibited CPT while C_6_-ceramides rapidly inhibited both CPT and EPT [46]. It is therefore more accurate to refer to ICL-CCIC-0019 as CDP-choline pathway inhibitor rather than a CHKA inhibitor as it inhibits the pathway at the commitment step and last step.

Further to the effect on substrate incorporation into PC, we also revealed a previously unappreciated effect of chemically inhibiting CDP-choline pathway activity on mitochondria function. Comparative metabolomics demonstrated that CHKA inhibition leads to a metabolically stressed phenotype analogous to mitochondria toxin treatment at 24 h after ICL-CCIC-0019 treatment. This drug-induced phenotype involved profound block of the TCA cycle characterized by a large decrease in steady state levels of mitochondria enzyme-related metabolites including citrate and cis-aconitate, whereas α-ketoglutarate only decreased at the higher concentration. Conversely, glycolysis was increased after short-term incubation with ICL-CCIC-0019 (increased ECAR) accompanied by elevation of glycolysis-related metabolic intermediates including fructose-1,6-bisphosphate, glucose-6-phosphate, dihydroxyacetone phosphate and serine to provide important substrates to overcome the TCA cycle block. Tracing studies using ^13^C-glucose and [^18^F]-FDG further confirmed the increased glycolysis in the context of higher glucose uptake and quantitative lactate secretion, together with increases in glycolytic intermediates (increases in glucose-6-phosphate, glyceraldehyde-3-phosphate, serine), and TCA block (decreased flux into citrate and decreased glutamine uptake). A study evaluating the metabolite profile of cells undergoing etoposide-induced apoptosis [47] reported increases in glucose-6-phosphate, glyceraldehyde-3-phosphate and fructose 1,6 bisphosphate with apoptosis; whether our findings are linked to apoptosis requires further work. In general, fluxes into long-chain saturated fatty acids increased after treatment, while long-chain unsaturated fatty acids decreased; concomitantly, mitochondrial fatty acid-β-oxidation appeared blocked. We envisaged that TCA cycle block would limit synthesis of fatty acid from glucose carbons while fatty acid synthesis from acetate may be unaffected. By tracing [^14^C]-acetate, we showed that acetate uptake was increased in a dose dependent manner. ACC1 and FASN are cytoplasmic enzymes that can convert acetyl-coA to malonyl-coA and further to fatty acids; ACC2, which can also catalyse conversion of acetyl-coA to malonyl-coA, is a mitochondrial enzyme. The increased [^14^C]-acetate may be a survival mechanism.

We show in this study that ICL-CCIC-0019 inhibits mitochondrial function and activates glycolysis. The two processes may be linked. Intracellular citrate concentrations (decreased by ICL-CCIC-0019) and ATP/AMP concentrations (determinants of AMPK activity) are potent modulators of the rate limiting glycolytic enzyme phosphofructokinase [48]. ICL-CCIC-0019 activated AMPK (measured as increased pACC and pT172 expression), which may have contributed to the activation of glycolysis. From the kinase screen, we know that the effect of ICL-CCIC-0019 on AMPK was not a direct one but rather via modulation of cellular (mitochondrial) dynamics and possibly downstream of changes in mitochondria membrane potential and profound reduction of mitochondria respiration. The decrease in mitochondrial respiration occurred rapidly, as early as 5 min after adding ICL-CCIC-0019 suggesting a possible direct interaction of drug with mitochondria. From the observations made in this study, other potential mechanisms that can generate the reduced mitochondrial respiration phenotype include enhanced ethanolamine pathway activity (increased PEth and CDP-Eth) [49] or reduction in PCho and PC/PE [50, 51] and its effect on mitochondrial membrane asymmetry and membrane potential. Mitochondria have a strict requirement for highly dynamic translocation of membrane lipids synthesized in the cytoplasm, golgi or endoplasmic reticulum to support membrane integrity [52]. Changes in mitochondrial membrane potential in the hindlimb muscle of *chkb*−/− mice can be rescued by injection of CDP-choline suggesting a genetic basis in appropriate model systems. Thus, we can surmise that glycolysis was activated to support the inability of mitochondria to synthesize ATP (mitochondria membrane potential is required for ATP synthesis) and may explain the overall lack of significant reduction in cellular ATP.

Another interesting phenotype observed following treatment with ICL-CCIC-0019 was loss of mitochondrial networks. This was concomitant with a decrease of mitochondrial mass, characterized by reductions in citrate synthase expression in the colon cancer cells. Citrate synthase is a mitochondrial DNA encoded gene product, whose expression is a marker of cellular mitochondrial content (mitophagy biomarker). Interestingly, the ICL-CCIC-0019-induced citrate synthase depletion occurred in both A549 wild-type and A549 *Rho* cells that had lost mtDNA indicating that the ‘mitochondria stress phenotype’ was not due to effects on mitochondria redox carriers (Complex I-IV) required for mitochondrial respiration. This was further supported by reduced aspartate levels (Supplementary Figure S3E), which is largely synthesized via mitochondrial glutamic-oxaloacetic transaminase (GOT2) [53]. There is evidence that AMPK - a sensor of energetics - couples nutrient sensing and environmental stress to mitophagy and cell survival via phosphorylation of ULK1/2 or hATG1 [54].

In summary, we report a new mechanism of action for CDP-choline pathway chemical inhibition by ICL-CCIC-0019. In this model, drug treatment elicits loss of mitochondria membrane potential and mitochondrial respiration. The mitochondrial stress that ensues is concomitant with increased glycolysis, a process that is associated with AMPK activation. These newly described properties of choline kinase chemical inhibitors need to be considered when rational combinations are proposed.

## MATERIALS AND METHODS

### Chemistry and radiochemistry

The syntheses of choline kinase inhibitors and [^18^F]-D4-FCH have recently been reported [23, 28, 55].

### Cell culture and transfections

A431, A549, Ishikawa, MCF-7, and MDA-MB-468 cells were maintained in DMEM (Sigma-Aldrich). A2780 and HCT116 were maintained in RPMI (Sigma-Aldrich). St-T1b cells and MCF-10A cells were maintained in DMEM/F12 (Life Technologies). Media was supplemented with 10% FBS (Lonza) and antibiotics (Life Technologies). Cells were cultured at 37°C in humidified atmosphere containing 5% CO_2_. Cell lines were authenticated by provider by short-tandem repeat analysis. No additional authentication of cells was done by the authors. CHKA expression was silenced by doxycycline-inducible shRNAs targeting CHKA (pTRIPZ CHKA shRNA clones V3THS_335370 and V3THS_335372; Dharmacon) or non-targeting control (Dharmacon). Viruses were packaged using Trans-Lentiviral shRNA Packaging System (Open Biosystems). Knockdown was induced by incubation with 0.5 μg/mL doxycycline for 5 days.

### Choline kinase activity, proliferation, apoptosis and reactive oxygen species (ROS) assays

Enzymatic kinase activity, [^3^H]choline to [^3^H]PCho conversion and growth inhibition assays was conducted as previously described [23]. [^3^H]choline incorporation into soluble and lipid fractions was determined by a pulse-chase experiment. HCT116 cells were seeded at a density of 3 × 10^5^ cell per well into 12-well plates and incubated overnight. Cells were treated with 1 or 10 μM ICL-CCIC-0019 for 1 h and pulsed in presence of inhibitor with 18.5 kBq/mL [^3^H]choline chloride in 500 μL for 1 additional h. Cells were briefly rinsed with RPMI and incubated with fresh, non-radioactive medium for 6 h. Metabolites were extracted as described elsewhere [23], but additionally the radioactivity of the choline-containing and chloroform (lipid-containing) fractions was measured. Uptake of radiolabeled metabolite-tracers was measured as described elsewhere [56]. Antiproliferative activity was measured after 72 h continuous exposure to inhibitors by sulforhodamine B (SRB) assay as described elsewhere [23]. Apoptosis was determined using Caspase 3/7 Glo assay (Promega) according to the manufacturer's instructions.

For ROS measurements, cells were seeded in 96-well plates and treated with indicated ICL-CCIC-0019 (10 μM), pro- and antioxidants (0.25% H_2_O_2_ and 5 mM N-acetyl cysteine, NAC; all from Sigma-Aldrich) for indicated times. C400 (Life Technologies) was added at a final concentration of 50 μM and incubated at 37°C for 15 minutes and fluorescence quantified.

### Western blotting (general)

Cells were lysed in radioimmunoprecipitation assay (RIPA) buffer containing protease and phosphatase inhibitors (all from Sigma-Aldrich). Equal amounts of protein (20 μg) were resolved on 4–15% mini-protean TGX gels (Biorad) and transferred to PVDF membranes (Trans-Blot Turbo Transfer Packs, Biorad). Membranes were blocked for 1 h in 5% milk in Tris-buffered saline or phosphate buffered saline containing 0.1% v/v tween-20 (TBST or PBST) and incubated with the following antibodies overnight at 4°C: Actin (Abcam, Cat. Nr.: ab6276), ATF4 (Cell Signaling, 11815), CHKA (Sigma-Aldrich, HPA024153), HSP70 (Cell Signaling, 4876), HSP90 (Santa Cruz Biotechnology, sc-69703), IRE1a (Cell Signaling, 3294), and Ubiquitin (Cell Signaling, 3936). Secondary HRP-conjugated mouse and rabbit antibodies (Santa Cruz Biotechnology, sc-2004 and sc-2005, respectively) were applied for 1 h at room temperature. Signals were visualized using Amersham ECL Western Blotting Detection Reagent (GE Healthcare) and Amersham Hyperfilm (GE Healthcare).

Native gels were run using blue native polyacrylamide gel electrophoresis (BN-PAGE) method [57]. Cell homogenates were obtained by addition of passive lysis buffer (Promega) containing protease and phosphatase inhibitors to cell pellets and incubation for 30 minutes on a tube rotor at 4°C. Samples were cleared by centrifugation for 10 minutes at 12 000 × g at 4°C. Samples were prepared by mixing 30 μg protein with an equal volume of 2 × sample buffer (62.5 mM Tris-HCl, pH 6.8, 25% (v/v) glycerol, 0.01% (w/v) bromophenol blue, 0.02% (w/v) Coomassi Blue G250; all from Sigma-Aldrich). As control, D49N CHKA2 [58] was either applied under native conditions (100 ng diluted to 10 μL in 100 mM Tris buffer pH 7.4 added to 10 μL 2 × sample buffer) or denatured using SDS [100 ng diluted in RIPA buffer, 1 × NuPAGE LDS sample buffer and 1 × sample reducing agent (both Life Technologies)] and incubated at 70°C for 10 minutes. Samples were resolved on 4–15% mini-protean TGX gels using 1 × native running buffer (25 mM Tris, 192 mM glycine, pH 8.3). The cathode buffer was additionally supplemented with 750 μL Coomassi Blue G250 [final concentration ca. 0.04% (w/v)]. Membranes were transferred and probed as described above.

### DNA cell cycle analysis

Supernatant and cells were harvested, washed and centrifuged. The cell pellet was resuspended it 1 mL ice-cold PBS and the suspension added to 9 mL 70% ethanol, while mixing gently. Cells were fixed for at least 2 hours at −20°C. Cells were centrifuged and rehydrated in 3 mL cold PBS for 15 minutes. Samples were stained in 500 μL buffer containing 100 mM Tris pH 7.4, 150 mM NaCl, 1 mM CaCl_2_, 0.5 mM MgCl_2_, 0.1% Triton X-100, 0.1 mg/ml RNase A, 50 μg/ml propidium iodide (all items from Sigma-Aldrich) at 37°C for 3 h protected from light. Data were acquired on a BD FACS Canto flow cytometer (BD Bioscience) and analyzed using FlowJo 7.6.4 software (Tree Star).

### Cell membrane integrity assay

Cell membrane integrity was assessed using a fluorescent kit measuring lactate dehydrogenase (LDH) release (CytoTox-ONETM, Promega) according to manufacturer's instructions.

### Immunofluorescence microscopy

HCT116 cells (3 × 10^4^) were seeded into 4-well chamber slides (BD Biosciences), cultured overnight and treated with 1 or 10 μM ICL-CCIC-0019 for 24 h or 2 μg/mL tunicamycin (Sigma-Aldrich) for 4 h. Cells were washed 2 × 5 minutes with PBS and fixed with 4% formaldehyde (Sigma-Aldrich) in PBS for 15 minutes at room temperature. Cells were washed 3 × 5 minutes with PBS and permeabilized with 100 μL 0.1% Triton X-100 in PBS for 10 minutes at room temperature. Cells were washed 3 × 5 minutes with PBS at room temperature and blocked with 1% BSA / 0.1% Triton in PBS (PBST-BSA) for 1 h at room temperature. Cells were incubated with CHOP primary antibody (Cell Siganling, 2895) at a dilution of 1:3000 in 100 μL/well PBST- BSA overnight at 4°C. The next day, cells were washed 3 × 10 min PBS and incubated with secondary Alexa fluor 594 goat anti-mouse IgG antibody (Life Technologies, Cat. Nr: A-21125) at a dilution of 1:400 in 100 μL PBST-BSA for 1 h at room temperature in the dark. Cells were washed 3 × 10 minutes with PBS actin fibers stained with Phallloidin Alexa Fluor 488 (Life Technologies, Cat. Nr: A12379) for 15 min at room temperature. Cells were washed 3 × 10 minutes with PBS and coverslips mounted using ProLong Gold Antifade Reagent containing DAPI (Invitrogen, Cat. Nr: P-36931). Images were acquired on an Olympus BX51 microscope and DP controller software version 2.1.

### Choline extraction and radio-HPLC

We determined the effect of ICL-CCIC-0019 on initial choline extraction (transport and phosphorylation; Supplementary Figure S2E–S2F) using a modification of previously described methods [59]. Instead of [^14^C]choline, we used the catabolically more stable choline analogue, [^18^F]-D4-FCH. MN58b and Hemicholinium-3 (HC-3) were used as controls. HPLC was performed using methods previously reported by us for this radiotracer [27].

### Metabolomics

Unlabeled metabolite analyses were conducted as previously reported [5]. HCT116 cells cultured overnight in 6-well plates were treated with vehicle, 1 or 10 μM ICL-CCIC-0019 for 24 h prior to extraction. For flux experiments, HCT116 cells were plated onto 6-well plates and cultured overnight. The cells were then grown in medium containing vehicle or 1 μM ICL-CCIC-0019 for 24 h. The medium was then replaced with that containing ^13^C metabolites (5 mM ^13^C_6_-glucose or 0.2 mM ^13^C_2_-acetate) and inhibitor for various indicated time points. Metabolites were extracted with a cold solution (−20°C) composed of methanol, acetonitrile, and water (5:3:2). The insoluble material was immediately pelleted in a cooled (0°C) centrifuge at 16,000 × *g* for 15 min and the supernatant was collected for subsequent LC-MS analysis. A ZIC-pHILIC column (4.6 mm × 150 mm, guard column 2.1 mm × 20 mm, Merck) was used for LC separation using formic acid, water, and acetonitrile as component of the mobile phase. Metabolites were detected using an Exactive mass spectrometer (ThermoFisher).

### Analysis of mitochondrial function

### Mitochondrial respiration

Extracellular acidification and oxygen consumption rates (ECAR and OCR) were measured in a XF24 extracellular flux analyzer (Seahorse XF24 Extra Cellular Flux Analyzer, North Billerica, MA, USA). Basic XF assay medium was prepared as previously described [60] supplemented with glucose, and the pH adjusted to 7.4 at 37°C. Indicated final concentrations of compounds were obtained by injecting appropriate amounts of compounds dissolved in DMSO or water and diluted in XF assay medium into ports. For ECAR and OCR measurements 40–50,000 HCT116 cells were plated in each well in RPMI overnight and the assay was carried out according to the XF24 assay protocol suggested by the manufacturer. Indicated data points were obtained after 3 min mixing and 2 min wait, followed by 3 min of data collection. Data were normalized to the basal values.

### Mitochondrial protein and phospho-protein expression

1.5–2.0 × 10^6^ HCT116 cells were plated on 10 cm plates in RPMI medium. After 24 h, cells were treated with indicated concentrations of CHK inhibitors for a further 24 h. Plates were then washed with ice cold PBS and cells were removed with 300 μL lysis buffer (50 mM HEPES, 150 mM NaCl, 1 mM EDTA, 0.2 mM DTT, 1% triton and phosphatase and protease inhibitors), syringed through a 25G needle and incubated on ice for 10 min. Cell lysates were centrifuged at 14000 rpm for 30 min and the total extracts were collected and stored at −80^°^C. About 60 mg of cell extracts were used for western blot analysis. Antibodies used for western analysis include, from Millipore, total *acetyl*-*CoA carboxylase* (ACC; 05-1098); from Cell Signalling, phosphorylated-ACC (pACC-S76; CS3661L), AMPK anti-pT172 (CS2535L), AMPK anti-B1 and B2 (CS 4150); from Abcam anti-cytochrome C oxidase subunit II (MTCOX-2; ab110258), anti-citrate synthase (CS; ab96600) and; from Santa Cruz Biotech, anti-GAPDH (FL-335). Antibody dilutions used were as suggested by the supplier.

### Mitochondrial immunofluorescence

20,000 HCT116 cells were plated on glass coverslips. After 24 h, cells were treated with 5 μM ICL-CCIC-0019. After 24 h cells were fixed with 1% paraformaldehyde for 20 min and blocked with fish gelatin overnight. Blocked cells were incubated with antimitochondrial antibody (Abcam; ab110413, 1:200 dilution) for 8–10 h. Coverslips were further washed with PBS and mounted with vector shield and DAPI, and imaged on a SP5 confocal microscope (Leica).

### TMRE-Membrane potential assay

HCT116 cells were cultured as above with indicated inhibitors and subject to the tetramethylrhodamine ethyl ester dye method (TMRE) membrane potential assay according to the manufacturers instruction (Abcam ab113852) prior to FACS analysis.

### Animal models

All animal experiments were conducted in accordance with the United Kingdom Home Office Guidance on the Operation of The Animals (Scientific Procedures) Act 1986 Amendment Regulations 2012 and within the published guidelines for the welfare and use of animals in cancer research [61]. Xenografts were established in female BALB/c nude mice aged 6–8 weeks (Harlan) by subcutaneous (s.c.) injection of 100 μL HCT116 cells (5 × 10^6^ in PBS) on the back of mice. Tumor dimensions were frequently measured by calliper and volumes calculated by the following equation: volume (mm^3^) = (π/6) × a × b × c, where a, b, and c represent 3 orthogonal axes of the tumor. When tumor volumes reached approximately 50–100 mm^3^ (ca. 10 days post injection), mice were used for subsequent studies.

### Pharmacokinetic and plasma metabolite studies

Plasma pharmacokinetic parameters of ICL-CCIC-0019 were established in female BALB/c mice aged 6–8 weeks (Harlan). Mice were intraperitoneally (i.p.) or perorally (p.o.) administered a single dose of 10 mg/kg and blood collected by cardiac puncture after 5, 15, 30 minutes and 1, 2, 4, 6 and 24 h. For tissue pharmacokinetic studies, HCT116 xenograft bearing mice were administered 10 mg/kg ICL-CCIC-0019 i.p. and blood collected after 5 minutes, 2, 6 and 24 h. Plasma was obtained by centrifugation at 1000 × g for 10 minutes. Samples were snap-frozen and stored at −80°C prior to analysis. Analysis was carried out by Cyprotex Inc. using liquid chromatography–mass spectrometry (LC–MS). For analysis of plasma metabolites, 2-hour samples of tissue pharmacokinetic studies were pooled.

### PET-CT imaging

Mice were anesthetized through isoflurane inhalation and scanned on a Siemens Multimodality Inveon small animal PET-CT scanner for [^18^F]-D4-FCH PET studies using HCT116 xenografts. Low dose CT scans were first acquired (80 kVp, 0.5 mA, 220 degree rotation, 600 ms per degree exposure time, 80 μm reconstruction pixel size) for PET attenuation correction and anatomical reference. PET images were acquired following a bolus i.v. injection of approximately 3.7 MBq tracer in the tail vein. Dynamic emission scans were acquired in list mode format over 60 minutes. Data were sorted into 0.5 mm sinogram bins and 19 time frames for image reconstruction by filtered back projection (4 × 15 seconds, 4 × 60 seconds and 11 × 300 seconds). The Siemens Inveon Research Workplace software was used for visualization of radiotracer uptake. To define 3-dimensional regions of interest (ROI), 30 to 60-minute cumulative images of the dynamic were employed. Arterial input function was estimated by drawing ROIs over the center of the heart cavity using cumulative images from 0.25 to 2 minutes of the dynamic series, a method previously validated for use in rodents [62]. The count densities were averaged for all ROIs at each time point to obtain time versus radioactivity curves (TAC). Tumor TACs were normalized to injected dose measured by a VDC-304 dose calibrator (Veenstra Instruments) and normalized uptake was expressed as percentage injected dose per mL tissue (NUV; %ID/mL).

### Kinetic modeling of [^18^F]-D4-FCH data

Kinetic analysis of PET data was performed applying a standard two-tissue irreversible compartmental model described in detail elsewhere [28].

### TCGA data analysis

The results shown here are in whole based upon data generated by the TCGA Research Network: http://cancergenome.nih.gov/ and were analyzed using http://www.cbioportal.org and http://firebrowse.org.

### Statistical analysis

Data were expressed as mean ± standard deviation (SD). Unless otherwise specified, the significance of comparison between two data sets was determined using unpaired, two-tailed Student's *t* test (GraphPad Prism version 5.1) and *P* < 0.05 defined as significant.

## SUPPLEMENTARY MATERIALS FIGURES AND TABLES


