# Supplementary material for: The novel choline kinase inhibitor ICL-CCIC-0019 reprograms cellular metabolism and inhibits cancer cell growth
Source: Oncotarget. 2016 May 19;7(24):37103–20. doi: 10.18632/oncotarget.9466 (PMC5095062; doi:10.18632/oncotarget.9466)
Supplement: Supplementary file 1 [file oncotarget-07-37103-s001.pdf]

The novel choline kinase inhibitor ICL-CCIC-0019 reprograms cellular metabolism and inhibits cancer cell growth

Supplementary Materials

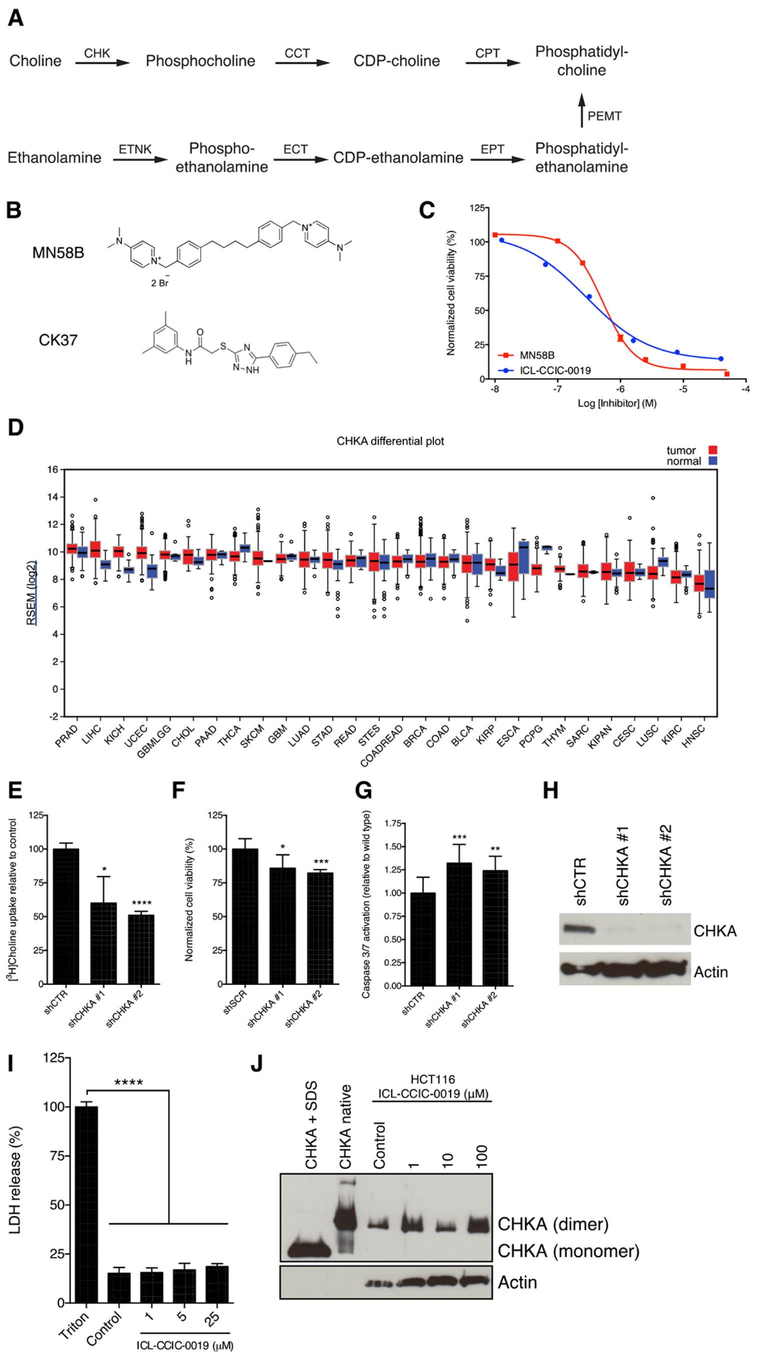

Supplementary Figure S1: (**A**) CDP-choline and ethanolaminekinase pathways of phosphatidylcholine (PC) and phosphatidylethanolamine (PE) synthesis. CHK, choline kinase alpha or beta; CCT, CTP:phosphocholine cytidylyltransferase; CPT, CDP-choline:1, 2-diacylglycerol cholinephosphotransferase; ETNK, ethanolamine kinase 1 or 2; ECT, CTP:phosphoethanolamine cytidylyltransferase; EPT, Ethanolamine phosphotransferase; PEMT, Phosphatidyl ethanolamine methyltransferase. (**B**) Structures of MN58B and CK37. (**C**) Comparative antiproliferative activities of ICL-CCIC-0019 and MN58B in A549 cells. [^{3}H] choline uptake (**D**),

CHKA mRNA expression in tumor vs. normal tissue across various cancer types (data: TCGA, analyzed via firebrowse.org). BLCA, Bladder urothelial carcinoma; BRCA, Breast invasive carcinoma; CESC, Cervical and endocervical cancers; CHOL, Cholangiocarcinoma; COAD, Colon adenocarcinoma; COADREAD, Colorectal adenocarcinoma; ESCA, Esophageal carcinoma; GBM, Glioblastoma multiforme; GBMLGG, Glioma; HNSC, Head and Neck squamous cell carcinoma; KICH, Kidney Chromophobe; KIPAN, Pan-kidney cohort (KICH+KIRC+KIRP); KIRC, Kidney renal clear cell carcinoma; KIRP, Kidney renal papillary cell carcinoma; LIHC , Liver hepatocellular carcinoma; LUAD, Lung adenocarcinoma; LUSC, Lung squamous cell carcinoma; PAAD, Pancreatic adenocarcinoma; PCPG, Pheochromocytoma and Paraganglioma; PRAD, Prostate adenocarcinoma; READ, Rectum adenocarcinoma; SARC, Sarcoma; SKCM, Skin Cutaneous Melanoma; STAD, Stomach adenocarcinoma; STES, Stomach and Esophageal carcinoma; THCA, Thyroid carcinoma; THYM, Thymoma; UCEC, Uterine Corpus Endometrial Carcinoma. (**E**) Cell viability (**F**), caspase 3/7 activity (**G**) and western blot against CHKA (**H**) upon 5 day treatment with doxycycline to induce CHKA shRNA in HCT116 cells. E-G, mean of $n = 3 \pm$ SD; $*P \leq 0.05$, $**P \leq 0.01$, $***P \leq 0.001$, $****P \leq 0.0001$. (**I**) Cell membrane integrity analysis measuring LDH release to the supernatant in HCT116 cells upon treatment with indicated doses of inhibitor for 4 h; mean of $n = 3 \pm$ SD; $****P \leq 0.0001$. (**J**) Maintenance of CHKA dimer integrity in HCT116 cells was determined by BN-PAGE upon treatment with indicated concentrations for 24 hours.

A

Mass	Metabolite name	Formula	Mass difference from parent	m/z found	Ion	Retention time (min)	Metabolite reference	Abs area	Area percentage
412	Parent	$C_{26}H_{44}N_4$	-	206	$[M]^{2+}$	7,38	-	7,17E+07	41,3
246	Loss of 166	-	-166	246	$[M]^+$	2,58	M1	1,02E+07	5,88
246	Loss of 166	-	-166	246	$[M]^+$	2,73	M2	1,22E+07	7,03
305	Loss of 107	-	-107	305	$[M]^+$	2,78	M3	7,54E+06	4,35
305	Loss of 107	-	-107	305	$[M]^+$	3,45	M4	1,31E+07	7,55
338	Loss of 74	-	-74	338	$[M]^+$	3,54	M5	1,01E+07	5,82
538	Gain of 126	-	126	538	$[M]^+$	10,46	M6	1,12E+07	6,46
563	Gain of 151	-	151	563	$[M]^+$	7,47	M7*	3,74E+07	21,6

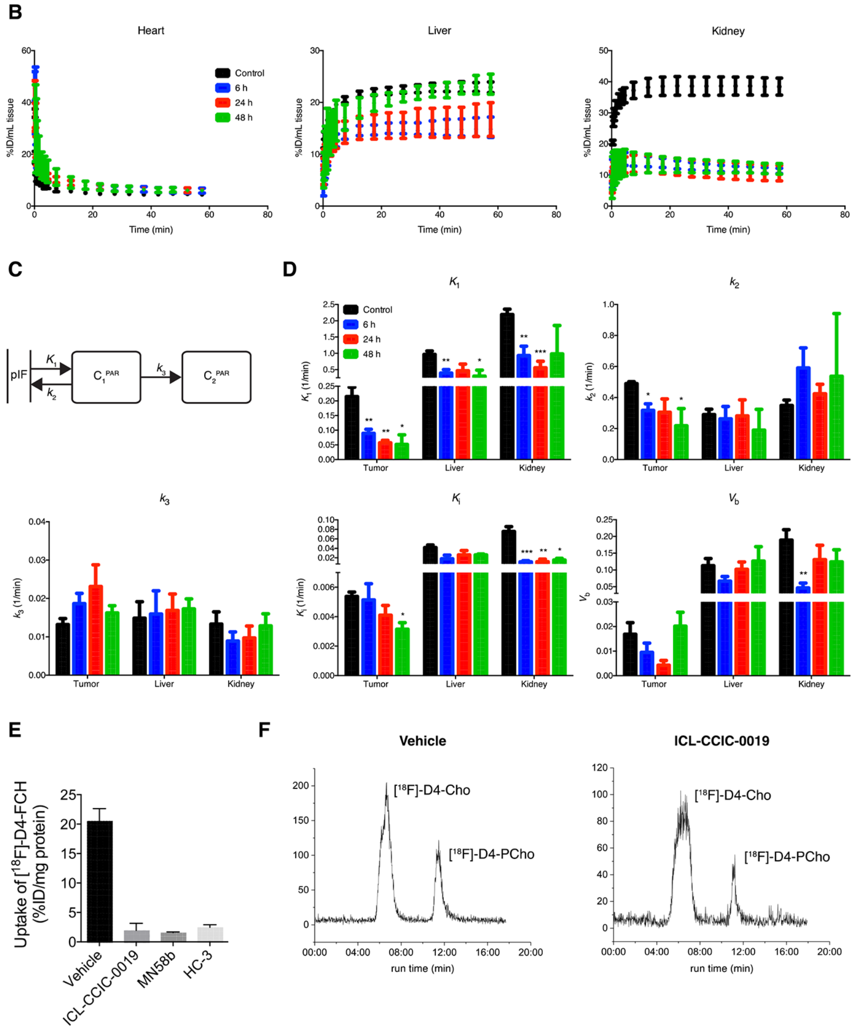

Supplementary Figure S2: (**A**) Plasma samples of BALB/c mice treated with 10 μM ICL-CCIC-0019 for 2 h used for tissue PK experiments were pooled and analyzed for parent peak and metabolites by LC-MS. (**B**) heart, liver and kidney TACs of [18F]-D4-FCH PET scans. (**C**) Scheme of two-tissue irreversible compartmental model. (**D**) Pharmacokinetic modeling of competitive inhibition of CHKA by ICL-CCIC-0019 using [18F] D4-FCH in HCT116 xenograft-bearing mice. Data represent mean of $n = 4$–5 per group ± SEM;

*$P \leq 0.05$, **$P \leq 0.01$, ***$P \leq 0.001$. (**E**) Competition assay based on the transport and phosphorylation of [^{18}F]-D4-FCH in the absence (vehicle; saline) or presence of choline kinase inhibitors (ICL-CCIC-0019 or MN58b) or choline transport inhibitor (Hemicholinium, HC-3) at 10 µM. HCT-116 cells were placed in Na-free buffer, and the buffer was replaced with one containing [^{18}F]-D4-FCH and inhibitor for 10 min. Data are mean ± SD, $n = 6$. (**F**) Radio-HPLC analysis of cells similarly treated with vehicle or 10 µM ICL-CCIC-0019 for 10 min. Note only parent [^{18}F]-D4-FCH and phosphocholine metabolite [^{18}F]-D4-PCho were identified on chromatograms; a betaine metabolite was not formed.

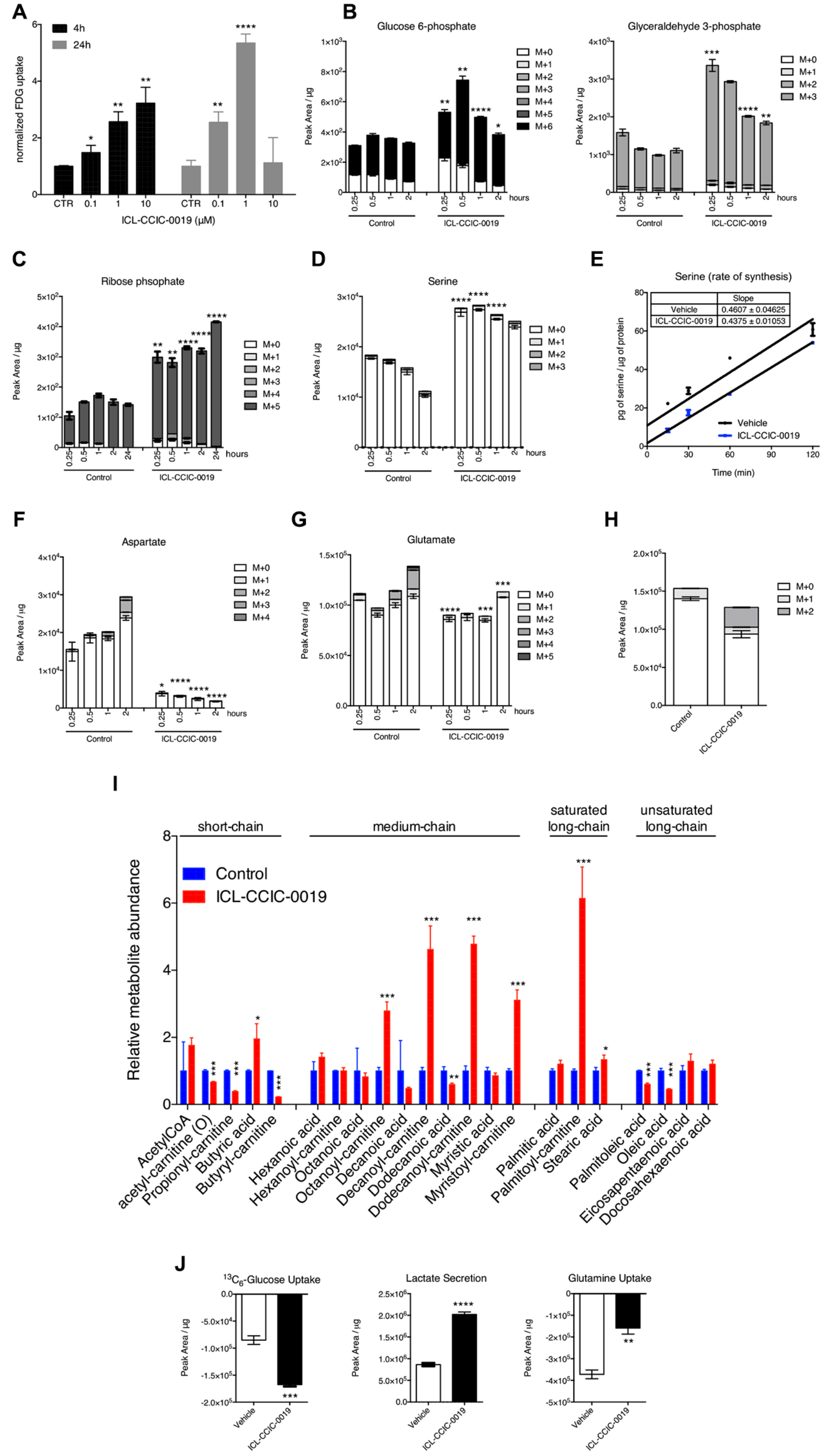

Supplementary Figure S3: (**A**) [^{18}F]-FDG uptake upon ICL-CCIC-0019 treatment in HCT116 cells. B-D, HCT116 cells were treated with ICL-CCIC-0019 for 24 h and subsequently subjected to ^{13}C-labeled glucose for indicated times. Intracellular glucose-6-phosphate, glyceraldehyde-3-phosphate (**B**), ribose phosphate (**C**), serine (**D**), rate of serine synthesis (**E**), aspartate (**F**) and glutamate (**G**) are shown. (**H**) Acetyl-carnitine from $^{13}C_2$-labeled acetate after 24 h of treatment with ICL-CCIC-0019. (**I**) Steady-state metabolomic changes in fatty acid components upon 24-hour ICL-CCIC-0019 treatment. (**J**) Transmembrane flux of glucose, lactate and glutamine. Mean of $n = 3 \pm$ SD; * $P \leq 0.05$; ** $P \leq 0.01$, ***$P \leq 0.001$, ****$P \leq 0.0001$.

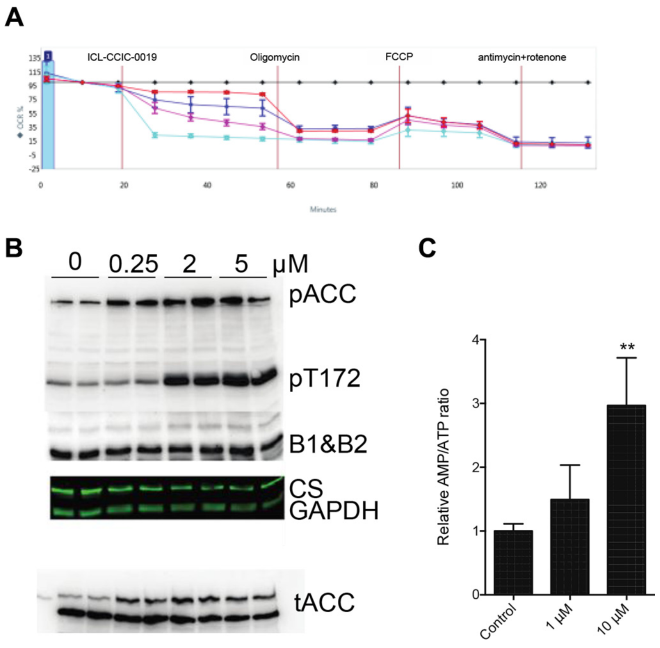

Supplementary Figure S4: (**A**) Effect of CHK inhibitors and AMP kinase activator on OCR. A, background (black), control (red), 10 µM AZD991 (blue), 10 µM MN58B (magenta), 10 µM ICL-CCIC-0019 (cyan). (**B**) Effect of ICL-CCIC-0019 on AMP kinase phosphorylation and citrate synthase as in Figure 6C with total ACC included. (**C**) relative AMP/ATP ratio as determined by LC-MS. Mean of $n = 4 \pm$ SD, $^{**}P < 0.01$.

Supplementary Table S1: Screen of ICL-CCIC-0019 against a panel of 131 recombinant kinases

Kinase	Species	Accession no.	% activity remaining	SD
ABL	human	NM_005157	112	5
AMPK	rat	Tissue purified	109	11
ASK1	human	NM_005923	119	5
Aurora A	human	BC027464	98	1
Aurora B	human	NM_004217	102	9
BRK	human	NM_005975	117	10
BRSK1	human	NM_032430	110	2
BRSK2	human	AF533878	94	3
BTK	human	NP_00052.1	131	1
CAMK1	human	NM_003656	110	11
CAMKKb	human	NM_153499	91	3
CDK2-Cyclin A	human	NM_001798/NM_001237	112	30
CDK9-Cyclin T1	human	NM_001261/NM_001240.2	95	7
CHK1	human	AF016582	97	4
CHK2	human	NM_007194	90	2
CK1γ2	human	BC018693.2	92	5
CK1δ	human	NM_001893	100	5
CK2	human	NM_001895	86	1
CLK2	human	NM_003993.2	101	1
CSK	human	NM_004383	96	1
DAPK1	human	NM_004938.2	96	13
DDR2	human	NM_006182	85	13
DYRK1A	human	NM_130437.2	114	1
DYRK2	human	NM_003583	96	2
DYRK3	human	AY590695	106	1
EF2K	human	AAH32665	99	11
EIF2AK3	human	NM_004836.5	97	1
EPH-A2	human	NM_004431	93	12
EPH-A4	human	NM_004438	124	10
EPH-B1	human	NM_004441	112	6
EPH-B2	human	NM_004442	90	1
EPH-B3	human	NM_004443	107	11
EPH-B4	human	NP_004435.3	110	5
ERK1	human	BC013992	91	1
ERK2	human	NM_002745	103	6
ERK8	human	AY065978	69	4
FGF-R1	human	M34641	92	11
GCK	human	BC047865	89	12
GSK3b	human	L33801	94	13
HER4	human	NM_005235	79	7
HIPK1	human	NM_198268	106	6
HIPK2	human	AF326592	104	3
HIPK3	human	NM_005734	112	35
IGF-1R	human	NM_000875	65	7
IKKb	human	XM_032491	101	8

IKKe	human	NM_014002	123	36
IR	human	NM_000208.2	103	16
IRAK1	human	NM_001569.3	89	12
IRAK4	human	BC013316.1	83	11
IRR	human	NM_014215	92	6
JAK2	human	NP_004963.1	90	4
JNK1	human	L26318	98	21
JNK2	human	L31951	100	15
JNK3	human	NM_002753	92	2
Lck	mouse	X03533	118	29
LKB1	human	NP_000446	100	7
MAPKAP-K2	human	NM_032960	89	4
MAPKAP-K3	human	NM_004635	67	6
MARK1	human	AF154845	103	8
MARK2	human	NM_004954	86	3
MARK3	human	U64205	106	5
MARK4	human	AK075272	95	4
MEKK1	human	XM_042066	92	1
MELK	human	NM_014791	106	14
MINK1	human	NM_015716	100	7
MKK1	human	L05624	82	0
MKK2	human	NM_030662	95	9
MKK6	human	NM_002758	102	2
MLK1	human	NM_005965	91	2
MLK3	human	NM_033141	110	4
MNK1	human	NM_002419	101	2
MNK2	human	AB000409	96	25
MPSK1	human	AF237775	105	5
MSK1	human	AF074393	96	2
MST2	human	CR407675	89	20
MST3	human	U60206	117	9
MST4	human	AAH65378	113	4
NEK2a	human	NM_016542	101	7
NEK6	human	NM_002497	86	6
NUAK1	human	NM_014397	103	20
OSR1	human	NM_014840	99	3
p38a MAPK	human	NM_005109.2	108	9
p38b MAPK	human	L35264	107	3
p38d MAPK	human	Y14440	100	2
p38g MAPK	human	Y10487	99	25
PAK2	human	Y10488	107	6
PAK4	human	NM_002577	114	5
PAK5	human	O96013	90	9
PAK6	human	Q9P286	107	7
PDK1	human	Q9NQU5	133	1
PHK	human	X80590	94	6
PIM1	human	NM_002613	102	8

PIM2	human	NM_002648	103	20
PIM3	human	U77735	113	2
PKA	human	Q86V86	106	2
PKBa	human	NM_002730	101	12
PKBb	human	BC000479	93	1
PKCa	human	NM_001626	98	3
PKCz	human	NM_002742	106	10
PKCγ	human	NM_002737	93	4
PKD1	human	NM_002739	105	5
PLK1	human	NM_002744	112	20
PRAK	human	NM_005030	101	11
PRK2	human	AF032437	94	13
RIPK2	human	S75548	102	0
ROCK 2	human	NM_003821	97	1
RSK1	rat	U38481	78	6
RSK2	human	NM_002953.3	105	10
S6K1	human	NM_004586	88	1
SGK1	human	NM_003161	94	14
SIK2	human	NM_005627	109	3
SIK3	human	NM_015191.1	102	16
SmMLCK	human	Sugen Kinase Database 15721	104	18
Src	human	NM_005417.3	105	1
SRPK1	human	NM_003137	106	3
STK33	human	BC031231.1	99	3
SYK	human	AAH01645.1	92	9
TAK1-TAB1	human	NM_003188 and NM_006116	97	2
TAO1	human	NM_020791	100	13
TBK1	human	NM_013254	108	20
TESK1	human	NM_006285.2	101	17
TIE2	human	BC035514.1	89	4
TLK1	human	NM_012290.4	110	20
TrkA	human	NM_001007792.1	84	15
TSSK1	human	AY028964	107	5
TTBK1	human	NM_032538.1	97	4
TTK	human	NM_003318	101	5
VEG-FR	human	NM_002019.3	80	6
WNK1	human	NM_018979.3	102	2
YES1	human	NM_005433	110	4
ZAP70	human	NM_001079	102	5
